# Design and validation of a cross-cultural virtual exchange experience among bilingual medical students

**DOI:** 10.3389/fmed.2025.1339277

**Published:** 2025-02-26

**Authors:** John R. Vaile, Cierrah Doran, Arlene R. Maheu, Valentina Jaramillo, Daniela A. Perez-Chadid, Tomas Fernandez, Carissa C. Walkosak, Dinah Roll Velez, Sara Carolina Bedoya Osorio, Courtney Seebadri-White, Susan Truong, Dimitrios Papanagnou, Andres Fernandez, Rosemary Frasso

**Affiliations:** ^1^Sidney Kimmel Medical College, Thomas Jefferson University, Philadelphia, PA, United States; ^2^College of Population Health, Thomas Jefferson University, Philadelphia, PA, United States; ^3^Escuela de Medicina, Universidad CES, Medellín, Colombia; ^4^Asano-Gonnella Center for Research in Medical Education and Health Care, Sidney Kimmel Medical College, Thomas Jefferson University, Philadelphia, PA, United States

**Keywords:** bilingualism, virtual learning, case-based learning, global health, medical education

## Abstract

Exposure to cross-cultural medical education is essential for professionals aiming to work in global health or serve diverse communities. However, traditional in-person exchange programs can be complex, costly, and restricted, particularly during times when travel is limited, such as during the COVID-19 pandemic. These challenges prompted us to explore novel approaches to support cross-cultural exchange programs. We designed, piloted, and evaluated a cross-cultural virtual exchange experience using case-based learning (CBL). Our study involved 14 bilingual medical students from the United States and Colombia who participated in four virtual CBL sessions. Following each session, participants engaged in guided discussions to reflect on their attitudes toward the experience, and content analysis was framed by sociocultural learning theory (SCLT). This study provides justification and operational guidance for implementing and improving upon cross-cultural virtual exchange experiences among medical trainees, with potential applicability to other contexts.

## Introduction

Amidst today’s landscape of medical education, global health exposure has gained prominence (1–3). Despite the recognized merits of traditional in-person exchanges, financial and systemic barriers create exclusivity, and are compounded by logistical limitations (1, 2, 4). These challenges often limit participation to those with substantial resources and create disparities in access to global health education (2, 4). However, the extent to which virtual platforms can foster longitudinal engagement and high-quality educational experiences for medical students remains underexplored. Early observations indicate that virtual exchanges may broaden accessibility and offer varied learning opportunities (5), yet their capacity to fully replicate the benefits of in-person experiences requires further investigation.

For medical trainees, quality exchange programs involve a bidirectional experience that enable authentic cross-cultural learning (1–4, 6, 7). More specifically, this type of experience exposes learners to different healthcare settings, which can effectively strengthen clinical and communication skills along with self-confidence as a practitioner (1). Additionally, trainees who participate in exchange programs endorse higher levels of cultural sensitivity, heightened interest in community and public health, and an increased commitment to underserved populations (2, 4, 6). However, access to these opportunities is far from universal. Financial barriers related to travel, housing, and cost of living expenses in the host country may mean the experience is not feasible for all learners who are interested in participating (2, 3). Further, exchanges are too often unidirectional, as institutions in under-resourced settings may not be able to provide support to the trainees who would benefit from an exchange experience. This relationship is especially true in the context of exchanges between learners from lower-income and higher-income countries (2, 3).

While the COVID-19 pandemic led universities to temporarily suspend preexisting exchange programs (5, 8), it served as a catalyst for considering novel ways to advance sociocultural learning without requiring travel. Virtual platforms, a common way to deliver education in the pandemic era, had some limitations but proved to be a low-cost and largely effective way to connect and collaborate with learners and colleagues near and far (9, 10). Video conferencing tools like Zoom*™* became integral to virtual learning, enabling real-time interactions and collaboration across diverse geographical locations. Facilitating a cross-cultural virtual exchange experience via this type of platform greatly minimizes costs associated with exchanges, provides increased access to learners, and enables longitudinal engagement and bidirectionality. In this study, we aimed to qualitatively analyze a cross-cultural virtual exchange experience among bilingual medical students from the United States and Colombia using sociocultural learning theory.

## Literature review

### Case-based learning in medical education

Pedagogical innovation in medical education is continuously evolving to enhance learning outcomes and equip medical trainees with requisite knowledge to succeed in healthcare professions. Among the various teaching approaches, case-based learning (CBL) has gained prominence and is being used across many institutions for its effectiveness in promoting critical thinking, clinical reasoning, and problem-solving skills for learners (11, 12). Furthermore, over the last decade, many studies have validated the benefits of CBL in medical education (13). Firstly, CBL facilitates the integration of basic science knowledge with clinical practice by presenting real-life patient scenarios, thereby bridging the gap between theoretical learning and practical application (11, 14). Secondly, CBL promotes active engagement and participation among students, and fosters collaborative learning via dynamic peer-to-peer interaction (12, 13). Thirdly, CBL supports the development of clinical reasoning skills as learners discuss and analyze cases, identify relevant information, and formulate diagnostic and management plans (11, 13). Even more, some studies argue that CBL cultivates lifelong learning habits by emphasizing self-directed inquiry and up-to-date use of primary literature (12, 13). In this study, we chose to structure the cross-cultural virtual exchange experience around CBL because it is centered around peer-to-peer interaction, which was considered ideal for the purpose of our study.

### Bilingual learners in virtual spaces

Few studies have discussed the relationship between virtual learning and bilingualism. However, the studies that have explored this relationship frequently cite the importance of language and cross-cultural information sharing, and how these components advance the educational experience (15–18). For example, the Community of Bilingual English-Spanish Speakers Exploring Issues in Science and Health (CBESS) program was adapted to a virtual format during the COVID-19 pandemic and incorporated bilingual mentors and focused curricular content to maximize engagement while addressing the sociocultural needs of participants (15). This program combined synchronous activities, recorded presentations, and hands-on science kits delivered to learners’ homes. It resulted in improvements in learners’ science knowledge and interest in STEM careers through interactive and culturally relevant sessions (15).

Similarly, virtual nutrition and exercise classes by the Keck School of Medicine at the University of Southern California demonstrated the effectiveness of incorporating a bilingual format alongside culturally relevant information (16). These sessions, conducted in both English and Spanish, overcame barriers related to access such as childcare and transportation, and ultimately this format made health education more accessible to low-income, predominantly Spanish-speaking communities. Participants reported an appreciation for the bilingual format, which enhanced their understanding and engagement (16). In a similar study by Ottesen et al., a virtual conference in Haiti used a bilingual format to facilitate cross-cultural exchanges related to orthopedic knowledge; it showed high satisfaction and positive learning outcomes (17). Since virtual learning and bilingualism are two central pillars of our study, we sought to explore what types of interactions resulted among bilingual CBL participants, and then qualitatively analyze those interactions using sociocultural learning theory.

### Sociocultural learning theory

Sociocultural learning theory (SCLT) is a framework rooted in the interplay of social interaction, language, and collaborative learning processes. SCLT underscores the critical role of social interaction in the learning journey and emphasizes that individuals construct knowledge through active engagement with others (19). This relates to the significance of peer interactions, mentorship, and communal learning experiences in shaping cognitive development (19). Furthermore, language is deemed a fundamental tool for mediating learning within the SCLT framework. Language not only serves as a means of communication but also as a way to assign meaning, express thoughts, and internalize cultural practices and norms (19). Through language, learners engage in collaborative discourse, scaffold their understanding, and co-construct knowledge with others (19). Lastly, SCLT argues the concept of the Zone of Proximal Development (ZPD), which is the difference between a learner’s current level of competence and their potential for growth with appropriate support (19). Within the ZPD, learning is optimized through interactions with more knowledgeable peers who provide a scaffold to facilitate the learner’s progression toward higher levels of understanding or skill acquisition (19). This framework not only elucidates the dynamic nature of learning but also provides a comprehensive lens through which all content analysis for this study could be conducted. Ultimately, study authors decided on SCLT for content analysis because it captures the social, linguistic, and collaborative dimensions of the learning experience.

## Materials and methods

### Study setting

The study was approved by TJU’s Institutional Review Board (IRB) with parallel approval from Universidad CES’s Ethics Board and followed the Consolidated Criteria for Reporting Qualitative Research (20). We conducted a virtual exchange experience using a video conferencing platform (Zoom™) as an iteration of an in-person exchange, which was paused due to COVID-19, between Sidney Kimmel Medical College at Thomas Jefferson University (TJU) in the United States and Universidad CES in Colombia.

Case-based learning (CBL) was the mode of instruction for the virtual exchange experience. CBL is a cornerstone of the medical education curriculum at TJU, and it was selected because it is designed to foster collaboration between learners. Furthermore, CBL has been validated as a pedagogically sound approach (11, 14).

### Research team

The study was performed by a multidisciplinary research team composed of TJU and CES faculty, including public health, medical education, and physician researchers, as well as medical students (3 from TJU and 3 from CES), and a master of public health student. Data collection and analysis were supervised by a qualitative and mixed-methods researcher, who has over 20 years of experience.

### Study design

**FIGURE 1:**
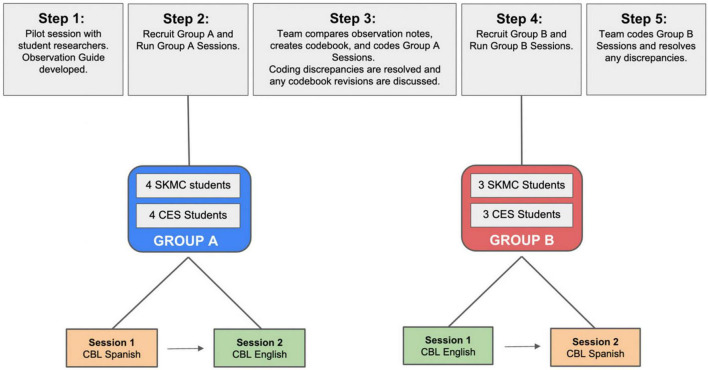
Structure of the cross-cultural virtual exchange experience.

The study team employed a qualitative observation approach to gather data during four virtual CBL sessions. Additional data were collected through observation of the student debriefing sessions that occurred immediately after the virtual CBL session was complete (Figure 1). Common observation approaches, such as structured observation through the development of a standardized observation guide, were adapted for the virtual setting. The study team met, discussed observation goals, and completed a pilot session to inform the observation guide that highlighted the types of actions and interactions that the team sought to document (21, 22).

Qualitative observation methodology was chosen as it permitted the research team to collect information about the interactions between participants during the exercise along with their behavior (8, 21). The study team chose the non-participant observation posture; in this context, that meant observers’ video cameras were disabled, their names were not visible, and observers did not contribute to the interactions during sessions or debriefing periods. However, participants were aware of their presence as it was disclosed during the consent process. The study team chose to utilize an observation approach followed by a participant debrief because this activity was new, and the study team sought to observe the exchange between learners. Future evaluations of this approach should include focus groups or 1:1 interviews with participants.

Medical student researchers from TJU and CES conducted the observations and participated in the coding and content analysis. During team meetings, time was dedicated to reflexivity and observers were given a chance to consider how their shared identity with the participants, who were also medical students, enhanced their ability to conduct a nuanced observation, and how this shared identity could bias their observations. On several occasions, medical student observers shared assumptions about the participants’ experiences based on how the observers thought they would act if in a similar situation. However, these assumptions were discussed and not allowed to inform analysis. Importantly, the study planning, logistics, and implementation were principally driven by the medical student members of the research team, with collaboration and direct supervision from the faculty members.

### Participant selection

In total, 14 students participated in this study (7 from each institution). At TJU, members of the Latino Medical Student Association (LMSA) chapter facilitated outreach for the recruitment of bilingual TJU students. At CES, the student research team members facilitated recruitment of bilingual medical students by word of mouth.

Participants were assigned to one of two groups based on availability. While all participants were in the later stages of the preclinical phase of medical school, it is important to note that the medical education system in Colombia differs from that of the United States. In Colombia, students matriculate to medical school after high school and complete a 6-year program. In the United States, students commonly complete a 4-year undergraduate degree before medical school, which takes an additional 4 years.

### Data collection

We used a video conferencing platform (Zoom*™*) to host and record four CBL sessions with students from TJU and CES. Two CBL cases were adapted by TJU faculty based on existing cases from the CBL case archive at TJU. One of the cases was translated into Spanish by a bilingual and native Spanish-speaking member of the team. CBL is a standard curricular element at TJU but not at CES, so before sessions began, the approach and expectations were explained in detail to all participants. The CBL cases used in this study had not been previously seen by participants.

After a pilot session among study authors, participants were divided into 2 groups (A and B). Group A was made up of four students from TJU and four from CES. Group B was made up of three students from each institution. Each group participated in two sessions: one in English and one in Spanish. Group A completed the Spanish session followed by the English session, and Group B completed the English session followed by the Spanish session. The first sessions were held in August and early September 2021 (Group A). The next two sessions occurred in late February and early March 2022 (Group B).

The pilot and each subsequent session were facilitated by a faculty member with extensive CBL experience. Each session lasted about 90 min and included a 10-min orientation, including obtaining verbal consent from each participant, about 1 h for case exploration, then a 20-min debriefing session. Debriefings were moderated by the senior author and conducted in English.

### Content analysis

The team deployed a directed content analysis approach (22). Codebook development was informed by initial observation notes, review of recordings of a pilot session among study authors only, and an exploration of the literature, with a focus on SCLT (Supplementary Appendix 1) (22, 23). In this context, guided problem-solving was achieved through faculty facilitation of the CBL process and through collaboration with peers. As mentioned, the central concepts of SCLT provided a lens through which content analysis was performed (19, 23).

After Group A completed two sessions, the observation guide and codebook were reviewed, and minor adaptations were discussed and approved by the research team before Group B sessions were conducted. While observers used an observation guide during each session, they viewed and coded the recordings later to ensure data analysis was comprehensive.

Each recording was coded by student members of the research team, all of which self-identified as bilingual in English and Spanish. Microsoft Excel^®^ was used to facilitate and organize the coding process. Coding accuracy was assessed by the senior author in consultation with a subgroup of the research team. All coding discrepancies were resolved via discussion. Observer notes, taken in real-time during each session, were not coded, but instead were used to inform discussions during team meetings. Additionally, the research team analyzed student reflections shared during the post-session participant debrief; these focused mostly on the structure and value of exchange programs and the virtual CBL experience.

## Results

We conducted qualitative observations of four cross-cultural virtual CBL sessions followed by facilitated debriefing sessions with participants. In total, 14 bilingual medical students (8 self-identifying as female, and 6 as male) participated in the sessions. During the CBL case exploration, participants worked together to solve the case (i.e., identify the correct diagnosis and treatment course).

Video recordings were coded and analyzed, and content analysis was organized into three concepts based on SCLT: (1) Cross-Cultural Information Sharing; (2) Translanguaging; and (3) Zone of Proximal Development. Each concept is discussed below and supported by participant quotations with translations in parentheses.

**Concept 1: Cross-Cultural Information Sharing**

During the debrief sessions, participating students discussed the importance of cross-cultural information sharing, noting that the experience provided them a chance to learn about medical conditions that are not common in their home country. This permitted learners the opportunity to observe and learn by seeing colleagues from other locations process information:

*“I think there is value in seeing these differences. I think it would be interesting to see a case that is not very common in the United States but is common in Colombia … [mainly] to see their train of thought*…” (CES student, Group A, Spanish session)

Participants shared that there are conditions and experiences that are common in Colombia but rare in the United States and vice versa. They explained that the experience prepared them to address cultural differences in their future practice of medicine.

*“I feel like [cultural competency] is so instrumental in our medical education. How can we expect to treat and help a diverse patient population if we are not mindful of the different backgrounds that we all come from?”* (CES student, Group B, English session).

**Concept 2: Translanguaging**

After reviewing recordings of the CBL process, the study team noted that, regardless of whether the case was presented in English or Spanish, the students invariably alternated between the two languages. For example, when a student sought the correct term to describe a symptom or treatment in the target language (i.e., the language in which the case was deployed), they looked to their colleagues for answers:

*“¿Cómo se dice poison ivy?” (“How do you say poison ivy?”)* (TJU student, Group A, Spanish session)

*“How do you say sarampión?” (“How do you say rubeola?”)* (CES student, Group A, English session)

Additionally, the team observed students acknowledging vocabulary shortcomings in their non-native language:

*“I was thinking of tos ferina, but I do not know how to say it in English.” (“I was thinking of pertussis, but I do not know how to say it in English.”)* (CES student, Group B, English session)

During the debrief sessions, participants discussed a mutual appreciation for the value of approaching a case in their non-native language along with a case in their native language using the support of classmates and native-speaking collaborators. One participant reflected on the benefit as it relates to their ability to communicate with future Spanish-speaking patients:

*“It was very helpful for me to not only hear but also try to explain certain things … If I have a Spanish-speaking patient [in the future], I will be able to explain [their diagnosis].”* (TJU student, Group B, Spanish session)

This sentiment was echoed by another student, who noted that there is a shared benefit among participants in learning the nuances of the other language, including sentence structure and word choice. On the other hand, some students shared that they were hesitant, especially at first, to contribute to the case discussion because they were worried about fluency in the non-native language. Further, some said that they often waited for “other people” to speak up:

*“For me, it was a little hard because I am not very good at English. I do not really have the confidence to speak, so I did not participate too much.”* (CES student, Group B, English session)

*“I feel like a lot of times if you do not speak the language well, but you are with other people who do, they can fill in the gaps for you and help make the conversation complete.”* (TJU student, Group A, Spanish session)

**Concept 3: Zone of Proximal Development**

The study team also observed peer teaching moments during each CBL session when participants would ask one another for additional information on a concept or term. For example, one TJU participant defined the term eczema in Spanish, then later provided its English translation along with a clinical correlate to inform a discussion of a patient with an asthma exacerbation.

*“Alguna gente que tiene eccema o inglés dice ‘atopic dermatitis’ tiene una … ¿cómo se dice predisposition? … predisposición para el asma, y es una reacción imunológica que puede causar una reacción en la piel pero también en los airways.”*

*(“Some people that have eczema, or atopic dermatitis in English, have a… How do you say predisposition?… predisposition for asthma, and it is an immunologic reaction that can cause a reaction in the skin but also in the airways.”* (TJU student, Group A, Spanish session)

Observation revealed that students were able to overcome all language challenges by working together with minimal or no support from the bilingual facilitator. During each session, students helped one another overcome language challenges by alternating between English and Spanish. They readily provided support to students from their home institution and partner institution.

During the debriefing sessions, several students noted that lectures and studying could be mundane, and virtual CBL was an alternative way for students to collaborate and learn together as they worked through a medical case. Specifically, one participant discussed the stepwise format of a virtual case and how it enables information sharing:

*“I like how it is built up in stages … getting to hear other peoples’ thoughts before heading to the next part.”* (TJU student, Group A, English session)

Similarly, participants agreed that working together through a CBL case is an attractive feature of the virtual session:

*“This is a structure you should follow when you are solving a case [at the clinic], so it also gave me the tools and mental structure to follow it [in the future].”* (CES student, Group A, Spanish session)

Students shared that being able to confidently converse in their non-native language was a hurdle that they had to overcome to maximize the experience. However, they shared that despite some language barriers, the virtual session allowed students to lean into the strengths of groupmates to meet an educational objective, whether it be understanding a medical concept, learning a new vocabulary word, improving insight into cultural differences, or more generally, strengthening interpersonal communication skills.

**Concept 4: Improving the Experience**

During the debriefing sessions, students were asked about ways the virtual experience can be improved. While students universally reported a positive experience and felt the format was effective and likely appealing to other students, several wished they had time to get to know their group before discussing the case. Some learners noted that they were more comfortable during the second session than the first.

*“I was more nervous [the first time]. But I guess the last time we did not know each other, so it was a little bit more difficult to begin the experience [during the first session].”* (CES student, Group A, English session)

Immediately after this reflection was shared, one student offered a solution:

*“Have breakout rooms … so students can intermingle with one another and talk through certain [concepts] … Then reconvene before each next step to share what each individual group [discussed].”* (TJU student, Group B, English session)

One participant suggested clearer instructions be given at the beginning of the session related to the expectations of the group:

*“Maybe give a disclaimer at the beginning: ‘For this exercise, we would like for you to utilize each other rather than external resources.”’* (TJU student, Group B, English session)

Lastly, while the participants believed there is value in a cross-cultural virtual exchange experience and stated that the session length was appropriate, they acknowledged that it would be challenging for medical students to commit to non-credit bearing or optional programming since their schedules are demanding, and they need to prioritize graded activities.

## Discussion

Cross-cultural exchange experiences offer medical students the potential for personal and professional development. However, in-person exchanges are not universally accessible, as there are often financial and logistical barriers. In contrast, virtual exchange experiences can offer learners the opportunity to engage in a collaborative activity with peers from another culture, country, or educational setting with no additional cost to the participant and minimal burden on the faculty.

In this study, we explored the value of a cross-cultural virtual exchange experience using SCLT. We conducted and observed four sessions with medical students from TJU and Universidad CES. Participants reported appreciation for the experience, noting that the virtual format provided an opportunity to learn from students from another culture. They shared that the experience informed their ability to care for their future patients in a way that is mindful of each culture. Specifically, all participants endorsed the feeling that they would be more comfortable speaking with future patients from cultures or countries different than their own.

Our observations of the CBL sessions and subsequent debriefs aligned with the concepts inherent in SCLT. For example, participants, in the absence of a direct question, appreciated the social interactions that evolved during the virtual CBL sessions and described the cross-cultural nature of the experience as valuable. Prior studies on cross-cultural exchanges indicate that these types of interactions can significantly enhance cultural competence and interpersonal skills among participants (15–17). More specifically, the experience provided a chance to appreciate how others process information and approach diagnostic challenges. Furthermore, they shared that it provided a conversational way to practice language skills and gave them insight into how important communication is in the work they do. Fung et al. concluded similar findings related to overcoming language barriers, and also reported a 15-percentage-point increase (from 64 to 79%) in pre- to post-test examinations on the session topics (18). Ultimately, learners who participated in virtual exchange experiences spoke positively about the value of collaboration; they discussed the benefits of learning from one another and co-creating opportunities to learn under the guidance of the CBL facilitator.

Additionally, participants were able to identify, dissect, and internalize these teaching moments with one another, and ultimately establish cross-cultural threads related to each country’s respective values and beliefs. Similarly, prior studies have concluded that bilingual learners in virtual spaces gain valuable insights into different medical practices and healthcare systems, and as a result, their global perspectives are enriched (15–18). Our study builds on these works by further characterizing the value of peer teaching. Traditional in-person exchange programs typically center on longstanding academic hierarchies like didactic sessions, trainees passively shadowing physicians, or dissemination of knowledge in a top-down manner—-from educator to trainees as opposed to among peers of similar educational levels (24). However, participants in our study recognized the value of a lower-stakes, inclusive, and accessible environment that still maintained high-quality educational objectives alongside active engagement and peer-generated teaching moments. Many agreed that this type of experience should be positioned alongside formal curricular offerings to diversify the scope of their global health exposure.

Apart from learning new medical cases, the participants engaged in another language. During the debrief sessions, participants considered this a worthwhile experience, specifically as it related to learning medical vocabulary, common phrases, and ‘slang’ from exchange partners. Multiple students mentioned that despite not having initial confidence in their fluency in the target language, their willingness to take a risk and attempt to find the correct word increased as students became more comfortable with peers. This type of interaction is supported by prior works discussing the concept of translanguaging, whereby bilingual learners utilize their full linguistic repertoire to enhance overall communication and learning for the group (9, 16, 18, 19, 25, 26). These benefits are particularly insightful since the cross-cultural virtual exchange experience can be adapted to other educational contexts.

Lastly, a cross-cultural virtual exchange experience is ethically compatible with the mission of global health (9). The framework of the virtual experience was developed to ensure that resources are appropriated such that participants, regardless of geographic location, income, education, or social capital, can access similar personal and academic enrichment opportunities. Considering this priority, a virtual experience functions to engage trainees with different cultures while being cognizant of logistical, financial, or even political barriers that may limit participation in this type of collaboration. The extent to which financial resources can be conserved is not within the scope of this study but may be a useful endpoint for future investigations.

### Limitations

This study has several limitations. First, the collaborating institutions had a preexisting and longstanding relationship that facilitated the operational and logistical aspects of the experience; it is possible that the approach and structure would be different in the setting of newly collaborating institutions. Second, we conducted only four virtual sessions, so it is possible that the experience could change over time in a longitudinal program with recurrent sessions. Additionally, the majority of participants had a sufficient level of fluency in their non-native language that allowed them to participate in the CBL activity and receive peer support when needed, but the experience might change if there is a large imbalance in language fluency among participants. Lastly, the sessions were anchored on CBL as the mode of instruction, but the experience may differ when implementing other types of educational activities.

## Conclusion

A cross-cultural virtual exchange experience among bilingual medical students from the United States and Colombia demonstrated significant educational value and potential for broader implementation. By employing case-based learning (CBL) in a virtual space, learners were able to engage deeply with peers from diverse backgrounds, and thereby enrich their understanding of the partner country’s cultural values, language, diagnostic methods, and treatment protocols for each CBL case. This process aligns with sociocultural learning theory (SCLT), which emphasizes the critical role of social interaction and collaborative learning in knowledge construction. The findings of this study are consistent with prior research indicating that virtual exchanges can effectively enhance cultural competence and interpersonal skills among medical trainees (1, 4, 6, 15–18).

Our study highlighted the importance of translanguaging, where participants utilized their bilingual abilities to navigate between languages and facilitate better communication and understanding (15, 17, 25). This method not only improved their language skills but also raised their confidence in using the non-native language in an educational space. The peer teaching and collaborative problem-solving observed during the sessions underscored the value of a lower-stakes, inclusive environment that promotes active engagement and mutual support among learners. These outcomes are supported by previous literature on bilingual education and virtual learning environments, which have demonstrated similar benefits in enhancing educational engagement and learning outcomes (15–18, 25, 26).

Despite the overall positive feedback, this study also identified areas for improvement, such as the need for more deliberate time for participant introductions and additional instructions about the CBL process. These insights are helpful for refining the structure of future virtual exchange programs. In sum, this study presents a proof-of-concept for a cross-cultural virtual exchange experience that can democratize access to global health education by minimizing financial and logistical barriers. Future research should focus on the long-term impacts of such exchanges and explore their applicability across different medical training contexts to further validate and expand upon these findings.

## Previous presentations

American Public Health Association Annual Conference on November 7, 2022 in Boston, Massachusetts, USA.

## Data Availability

The raw data supporting the conclusions of this article will be made available by the authors, without undue reservation.
