# Supplementary material for: Design and validation of a cross-cultural virtual exchange experience among bilingual medical students
Source: Front Med (Lausanne). 2025 Feb 26;12:1339277. doi: 10.3389/fmed.2025.1339277 (PMC11897259; doi:10.3389/fmed.2025.1339277)
Supplement: Supplementary file 1 [file Data_Sheet_1.docx]

Appendix 1: Codebook for the Cross-Cultural Virtual Case-Based Learning Sessions

| **Code Name** | **Definition** |
| --- | --- |
| Interactions Collaboration (Valence) | \| Use this code when TJU and CES have any inter-institutional communication, conversations, interactions, or collaborations, independent of facilitator prompting. Any type of communication, on any topic, between participants of different institutions that does not involve the facilitator. \|  \| \| --- \| --- \| |
| Interactions Parallel (Valence) | \| Use this code when the conversation is limited to one group (only between students from the same school), independent of facilitator prompting. Any type of communication, on any topic, between participants of the same institution that does not involve the facilitator. \|  \| \| --- \| --- \| |
| Language Clarification | Use this code when a participant asks a language clarification question, such as a translation or pronunciation request, or receives an unprompted clarification. |
| Case-Based Learning Question | \| Use this code when a participant asks a CBL process question. \|  \| \| --- \| --- \| |
| Direct Student Responses to Facilitator | \| Use this code when a participant responds to direct questions/prompting from the facilitator. \|  \| \| --- \| --- \| |
| Peer Teaching | \| Use this code when a participant explains a concept, idea, or medical knowledge to other participant(s), not including pure translation. \|  \| \| --- \| --- \| |
| Facilitator | \| Use this code when the facilitator directly prompts engagement. \|  \| \| --- \| --- \| |
| Stuck | \| Use this code when participants are not moving forward for some time (e.g., repeated questions from facilitator or participant on a single point). \|  \| \| --- \| --- \| |
| \| Breaking the Silence \|  \| \| --- \| --- \| | \| Use this code when the facilitator or participant breaks the silence in the conversation. \|  \| \| --- \| --- \| |
| Understanding Moments | \| Use this code when participants verbally acknowledge learning or understanding. \|  \| \| --- \| --- \| |
| Creating the Differential | \| Use this code when students have a conversation about the differential. \|  \| \| --- \| --- \| |
| Non-verbal | \| Use this code when participants use non-verbal cues. \|  \| \| --- \| --- \| |
| Aspects of CBL (Debrief) | Use this code when a participant describes the pros/cons of CBL. |
| Aspects of Language (Debrief) | Use this code when a participant describes the pros/cons of working through a case in their second language. |
| \| Aspects of Educational Platforms (Debrief) \|  \| \| --- \| --- \| | Use this code when a participant describes pros/cons or comparisons of small groups, lectures, CBL, etc. (NOTE: not including Zoom® vs. in-person) |
| Recommendations (Debrief) | \| Use this code for student-generated recommendations or negative feedback. \|  \| \| --- \| --- \| |
| Highlights (Debrief) | \| Use this code for positive student feedback. \|  \| \| --- \| --- \| |
| Collaboration (Debrief) | \| Use this code when students discuss collaboration broadly, such as its benefits and/or challenges. \|  \| \| --- \| --- \| |
| Of Interest (Debrief) | \| Use this code when there is something of interest not captured by the existing codes. \|  \| \| --- \| --- \| |
